# Novel Resilience Phenotypes Using Feed Intake Data From a Natural Disease Challenge Model in Wean-to-Finish Pigs

**DOI:** 10.3389/fgene.2018.00660

**Published:** 2019-01-08

**Authors:** Austin M. Putz, John C. S. Harding, Michael K. Dyck, F. Fortin, Graham S. Plastow, Jack C. M. Dekkers, PigGen Canada

**Affiliations:** ^1^Department of Animal Science, Iowa State University, Ames, IA, United States; ^2^Department of Large Animal Clinical Sciences, University of Saskatchewan, Saskatoon, SK, Canada; ^3^Department of Agriculture, Food and Nutritional Science, University of Alberta, Edmonton, AB, Canada; ^4^Centre de Développement du Porc du Québec Inc. (CDPQ), Québec City, QC, Canada

**Keywords:** resilience, disease resistance, feed intake, feeding duration, pigs

## Abstract

The objective of this study was to extract novel phenotypes related to disease resilience using daily feed intake data from growing pigs under a multifactorial natural disease challenge that was designed to mimic a commercial environment with high disease pressure to maximize expression of resilience. Data used were the first 1,341 crossbred wean-to-finish pigs from a research facility in Québec, Canada. The natural challenge was established under careful veterinary oversight by seeding the facility with diseased pigs from local health-challenged farms, targeting various viral and bacterial diseases, and maintaining disease pressure by entering batches of 60–75 pigs in a continuous flow system. Feed intake (FI) is sensitive to disease, as pigs tend to eat less when they become ill. Four phenotypes were extracted from the individual daily FI data during finishing as novel measures of resilience. The first two were daily variability in FI or FI duration, quantified by the root mean square error (RMSE) from the within individual regressions of FI or duration at the feeder (DUR) on age (RMSE_FI_ and RMSE_DUR_). The other two were the proportion of off-feed days, classified based on negative residuals from a 5% quantile regression (QR) of daily feed intake or duration data on age across all pigs (QR_FI_ and QR_DUR_). Mortality and treatment rate had a heritability of 0.13 (±0.05) and 0.29 (±0.07), respectively. Heritability estimates for RMSE_FI_, RMSE_DUR_, QR_FI_, and QR_DUR_ were 0.21 (±0.07) 0.26 (±0.07), 0.15 (±0.06), and 0.23 (±0.07), respectively. Genetic correlations of RMSE and QR measures with mortality and treatment rate ranged from 0.37 to 0.85, with QR measures having stronger correlations with both. Estimates of genetic correlations of RMSE measures with production traits were typically low, but often favorable (e.g., −0.31 between RMSE_FI_ and finishing ADG). Although disease resilience was our target, fluctuations in FI and duration can be caused by many factors other than disease and should be viewed as overall indicators of general resilience to a variety of stressors. In conclusion, daily variation in FI or duration at the feeder can be used as heritable measures of resilience.

## Introduction

Disease resilience can be defined as the ability to maintain relatively undiminished performance levels under infection (Albers et al., 1987; Doeschl-Wilson et al., 2012; Mulder and Rashidi, 2017). In the literature, much focus has been placed on separating disease resistance and tolerance (Bishop, 2012; Bishop and Woolliams, 2014; Lough et al., 2015). Disease resilience is an alternative to selection for a combination of resistance and tolerance (Guy et al., 2012; Mulder and Rashidi, 2017). Most studies on resilience (e.g., Mulder and Rashidi, 2017), however, consider only a single disease but an animal could be resistant or tolerant to one disease and more susceptible to other diseases. Currently, there are dozens of pathogens for swine around the world, including viral, bacterial, and parasitic infectious diseases (Zimmerman et al., 2012). Pathogens can be spread around the world. New pathogens and alternative strains will continue to develop as well. Breeding companies that market breeding stock across the globe have to simultaneously consider disease resilience to many of these pathogens and environments. Selecting animals that maintain performance in a typical commercial system provides a natural weighting of resilience to each disease based on the impact of each disease on productivity, along with the incidence or prevalence of the disease. van der Waaij et al. (2000) stated that observed production can be viewed as a selection index where the underlying components are weighted based on their impacts on performance. It is important, however, that the testing environment is representative of the target commercial environments. Resilience can be an effective, but “black-box” approach to selection for disease resistance and tolerance in animals (Mulder and Rashidi, 2017). One of the challenges, however, is to obtain heritable measures or indicators of resilience for selection, as elite breeding populations are typically kept in high-health conditions.

Recently, Elgersma et al. (2018) exploited routinely collected daily milk yield to quantify resilience in lactating dairy cows because daily milk yield is sensitive to diseases such as mastitis. Both significant drops in milk yield and day-to-day variation in milk yield within cow were used to quantify resilience. These phenotypes did not quantify disease resilience specifically, as it was not possible to validate that all changes in milk yield were related to infectious diseases. This becomes a multifactorial issue as causes for drops in milk yield can include mastitis, lameness, subclinical ketosis, and displaced abomasum, among others (King et al., 2018). This leads to these types of phenotypes capturing disease resilience along with general resilience (Elgersma et al., 2018). When selection for growth under a high stress environment was practiced in cattle, Frisch (1981) found that the selected animals were more productive under challenge but that this selection did not change their growth potential. If the goal is to only target disease resilience, this is a disadvantage for measuring production or deviations in production. For instance, in dairy cattle, using somatic cell count as an indicator trait may be better for selection against only mastitis than measuring productivity fluctuations in milk yield or feed intake. However, if the breeding objective is to maintain productivity regardless of the causes associated with milk yield deviations (i.e., general resilience), phenotypes that measure changes in productivity over time within animal are likely to have an economic value themselves (Elgersma et al., 2018).

Much is known about the relationship between feed intake (FI) and anorexia (Sandberg et al., 2006; Kyriazakis and Doeschl-Wilson, 2009). Production of cytokines such as interleukin-6 (IL-6) and tumor necrosis factor-alpha α (TNF-α) can cause a loss of appetite (Webel et al., 1997; Petry et al., 2007; Kyriazakis and Doeschl-Wilson, 2009). Knap (2009) suggested that individual day-to-day variation in feed intake could be utilized to quantify environmental sensitivity such as resilience to heat stress. Animals with more day-to-day variation in FI would indicate animals that are less resilient. Under a disease challenge, day-to-day variation in FI would reflect resilience to disease.

Alternative feed intake traits from individual FI electronic systems have been analyzed previously for the purpose of developing indicator traits for feed intake or feed efficiency in a selection index (de Haer et al., 1993; Von Felde et al., 1996; Schulze et al., 2003; Young et al., 2011; Lu et al., 2017). The most common and simplest of these traits investigated are occupation time at the feeder (or duration), number of visits, and FI rate (kg feed / unit time). Other feeding traits during the course of a day have also been investigated (Kyriazakis and Tolkamp, 2018). Individual FI is typically recorded in high-health environments, which limits the use of these data in nucleus herds to quantify traits related to environmental sensitivity or resilience (mostly due to health). FI traits such as feeding duration (i.e., time at the feeder) could also exhibit day-to-day variability from causes such as illness and may be a more feasible alternative to collecting individual FI in these challenged environments if typical commercial feeders could be enhanced with antennae to collect time at the feeder on individual pigs (with RFID tags). Feeding traits, such as duration, become more valuable in severely challenged environments due to the fact that if a pig stops eating completely their time at the feeder is expected to be zero.

The objectives of this study were to (1) develop and evaluate novel measures of resilience based on daily feed intake and feeding duration data for finishing pigs in a health-challenged environment and (2) determine heritabilities and genetic correlations of these measures with mortality, treatments, and other economically important production traits.

## Materials and Methods

This study was carried out in accordance with the recommendations of the Canadian Council on Animal Care (https://www.ccac.ca/en/certification/about-certification/). The protocol was approved by the Protection Committee of the Centre de Recherche en Sciences Animales de Deschambault (CRSAD; http://www.crsad.qc.ca/). The Centre de développement du porc du Québec (CDPQ) had full oversight on the project along with veterinarians.

### Natural Challenge Protocol

A natural challenge wean-to-finish protocol was established in late 2015 at CDPQ in Québec, Canada, with the aim to mimic a commercial farm with high disease pressure to maximize expression of genetic differences in resilience. The protocol was established at a research facility to allow detailed phenotype recording, blood sampling, and *in vivo* assays. This is an ongoing project that will conclude in early 2019. The natural challenge facility consists of three consecutive phases: (1) a healthy quarantine nursery for ~19 days after weaning, (2) a late nursery phase, where pigs are first exposed to disease for ~4 weeks, and (3) a finishing phase for the remainder of the growing period (69–181 days of age on average). Phases 2 and 3 are in the same barn, connected by a hallway and are collectively referred to as the “challenge facility.” Phase 1 is at a nursery approximately 1 km south of the challenge facility and is kept free of disease using strict biosecurity between the facilities. In the quarantine nursery, samples, and measurements are taken for future development of early predictors of resilience in a non-challenged environment, typical of a genetic nucleus. The number of pigs per pen is approximately four, seven, and thirteen for phases one to three, respectively. The quarantine nursery was not available for cycle 1 (first seven batches), for which phases 1 and 2 were combined. During this period, strict biosecurity was practiced between the nursery and finishing unit (same building connected by a hallway) but this was not sufficient to keep diseases from getting into the nursery, after which the quarantine nursery was established.

The natural disease challenge was established by bringing in naturally infected animals (seeder pigs) from strategically selected farms into the challenge barn (late nursery and finishing). Four groups of 12–28 pigs were introduced from three different commercial farms in the first four months of the study as seeder pigs. Thereafter, monitoring for diseases was focused on the test population and less on the seeder pigs. Initially, the targeted diseases included porcine reproductive and respiratory syndrome virus (PRRSV), porcine circovirus type 2 (PCV2), *Mycoplasma hyopneumoniae* (*M. hyo*.), *Actinobacillus pleuropneumonia* (APP), and swine influenza, and various opportunistic bacterial pathogens, including *Streptococcus suis* and *Haemophilus parasuis*. APP strain 12 was present. Three different strains of PRRSV present had ~85–90% sequence identity to the PRRS-MLV (Boehringer Ingelheim, St. Joseph, MO). Every batch was confirmed to have been exposed to PRRSV based on sampling a subset of individuals using PCR and serology four- and six-weeks post challenge, respectively. Multiple influenza subtypes were present in the barn including the H1N1 and H3N2 based on serological testing of a subset of the population at 18 weeks post entry. No typing for PCV2 or *M. hyo* was completed. The disease challenge was a function of these pathogens collectively in combination with the environment, management, and veterinary strategies designed to obtain a target infection pressure for each batch. The natural challenge was set up as a continuous flow system in order to maintain a steady health challenge without having to keep introducing pathogens, as well as for labor and flow considerations. A new batch of naïve pigs enters every three weeks and is generally provided fence-line contact with the preceding batch for ~1-week period, except during periods of excessively high infection pressure when it is discontinued to help reduce mortality rate to sustainable levels established by the Animal Protection Committee. For the data used in this study, the following viruses were identified in the challenge facility: PRRSV (3 strains), Influenza A virus of swine (AIV; 2 strains), porcine circovirus type-2 (PCV2), and porcine rotavirus A (RVA). Bacterial pathogens diagnosed included: *Actinobacillus pleuropneumoniae* (APP), *M. hyo., Streptoccus suis, Haemophilus parasuis, Brachyspira hampsonii, Salmonella sp., Cystoisospora suis* (*Coccidiosis), Ascaris suum, Erysipelothrix rhusiopathiae*, and *Staphylococcus hyicus* (causative agent for Exudative Epidermititis). Not all pathogens were identified in all batches, as would be the case on a commercial farm and other unidentified minor pathogens may also have been present. Although fairly endemic in the US, porcine epidemic diarrhea (PED) was not present in Québec and was therefore not present in the challenge facility.

To maintain acceptable levels of animal welfare and morbidity, individual treatments were given on a case-by-case basis, along with periodic batch-level (or mass) treatments. The treatment protocol was established by the consulting veterinarian, who is licensed in the province of Québec, Canada. Veterinarians had close oversight on the treatment protocol over time, which was adapted as needed to maintain acceptable levels of disease and minimize animal suffering. In addition, some treatment decisions were made by multiple veterinarians and trained barn staff, introducing some level of subjectivity, as would be the case in a commercial facility. Pigs exhibited clinical signs indicative of pneumonia, diarrhea, lameness, arthritis, meningitis, dermatitis, pallor, lethargy, weight loss, unthriftiness, cyanosis, or conjunctivitis. Pigs were treated with one of ten different antibiotics as per a regimented treatment protocol outlining primary and secondary (if needed) treatment choices for each ailment. For some clinical signs, one of two anti-inflammatory drugs were also administered. Batch-level water medication was used in the nursery when deemed necessary during periods of severe illness. One of two antibiotics were used in these batches. Furthermore, a water-soluble anti-inflammatory drug was also periodically administered in the nursery to treat batches that suffered from severe respiratory disease (primarily related to PRRSV infection). After the first seven batches, vaccination for PCV2 was added to the quarantine protocol in response to necropsy data linking characteristic lymphoid lesions with the presence of the virus. Reports from feed intake recording were generated daily for farm staff to identify sick pigs that did not eat as much as expected. Euthanasia decisions for animal welfare reasons were made by farm staff, with appropriate veterinary oversight. Barn air and temperatures were controlled with a ventilation system and a heater was used to regulate the lower bound temperatures within the barn.

A new batch of pigs entered the natural challenge protocol every three weeks. Each batch consisted of ~60 or ~75 weaned Large White by Landrace (or reciprocal mating) barrows (castrated male pigs) that were provided by one of the seven members of PigGen Canada (https://piggencanada.org/) from healthy multiplier farms. Each batch was sourced from one multiplier, but over time different multipliers could supply pigs for a given PigGen member. Variables collected on piglets at the multiplier farms were date of birth, wean age, and biological sow ID. The protocol specified that two to four weaned barrows should be sampled per litter. Eighty-seven percent of all piglets met that criterion. Piglets were retagged with a sequential ID tag when they arrived at the first nursery. Every seven batches were considered a cycle, numbered one to three in the current study. Each company was represented once each cycle (i.e., one batch per company per cycle). This continued for a total of three cycles, therefore each company was represented three times in the data analyzed here. This came to a total of 1,341 pigs that entered the facilities within the time period studied.

**Figure 1:**
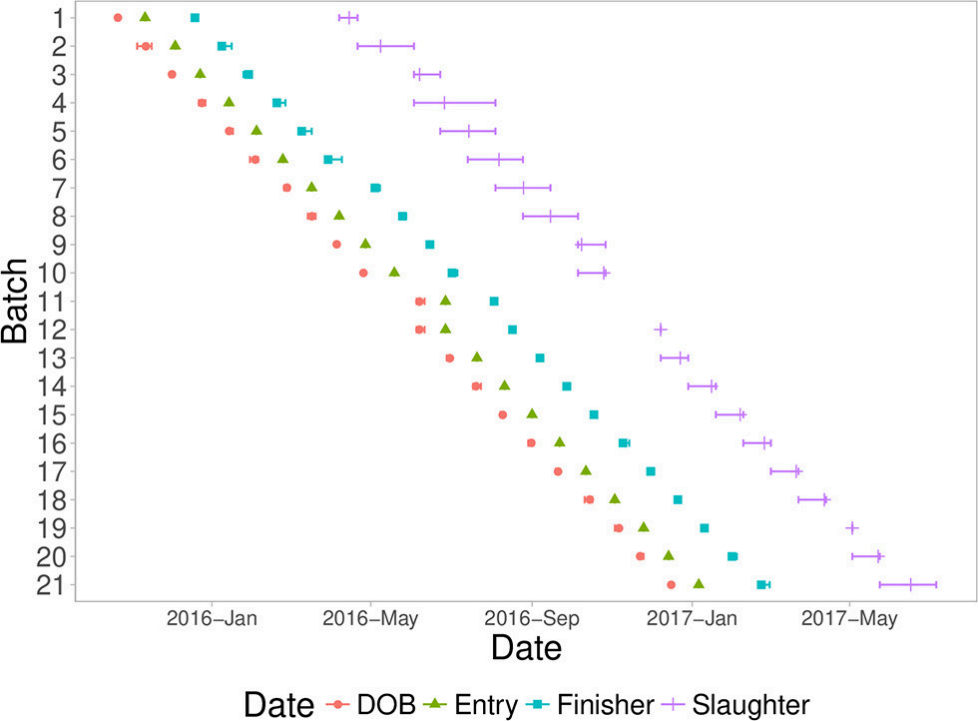
Timeline of the batches entered into the natural challenge facility, including mean (symbol) and min/max (error bars) of each batch with date of birth (DOB), date of quarantine nursery entry (Entry), date of finisher entry (Finisher), and date of slaughter (Slaughter).

A fixed weight system was used to identify pigs for slaughter, starting at ~180 days of age. Pigs that were not heavy enough were delayed for three weeks and then evaluated again. Most batches took between two to four slaughter groups to slaughter all pigs from a batch. Figure 1 shows a timeline of all batches analyzed in this study, with timing of date of birth, entry into the first nursery, entry into the finisher, and slaughter dates.

### Data

All data and samples were collected by trained research staff from CDPQ following established protocols. Body weights were taken approximately every three weeks. However, if a pig was unhealthy, it may have been weighed a few more times, closer together in time. To obtain daily weights, a LOESS (Locally Weighted Scatterplot Smoothing) regression was fit to all weights available for an animal, using the loess function in R using defaults (R core team, 2017). LOESS regression is a form of nonparametric regression, also known as local regression, that can fit non-linear trends in a flexible enough manner to “connect the dots” between weight measurements. The correlation of LOESS predicted with observed weights was 0.9995 for days with an observed weight. The LOESS predicted daily weights were utilized for calculations of production measures such as feed efficiency and growth (see below).

Feed intake data was recorded in the finishing phase using IVOG® feeding stations (Insentec, Marknesse, Netherlands). Feed was available *ad libitum* throughout the study. The nursery feeding protocol consisted of four diet phases, while the finishing period included two diet phases. Individual feed intake visits were processed and cleaned by CDPQ staff using the methods of Casey et al. (2005) and were aggregated into daily totals for each pig, including total amount of feed consumed (kg) and duration (time) at the feeder (minutes). Daily totals of more than 5 kg of feed were set to missing. Missing daily values were subsequently imputed using a 5-day rolling average within animal (also used if there were two adjacent days missing).

**Figure 2:**
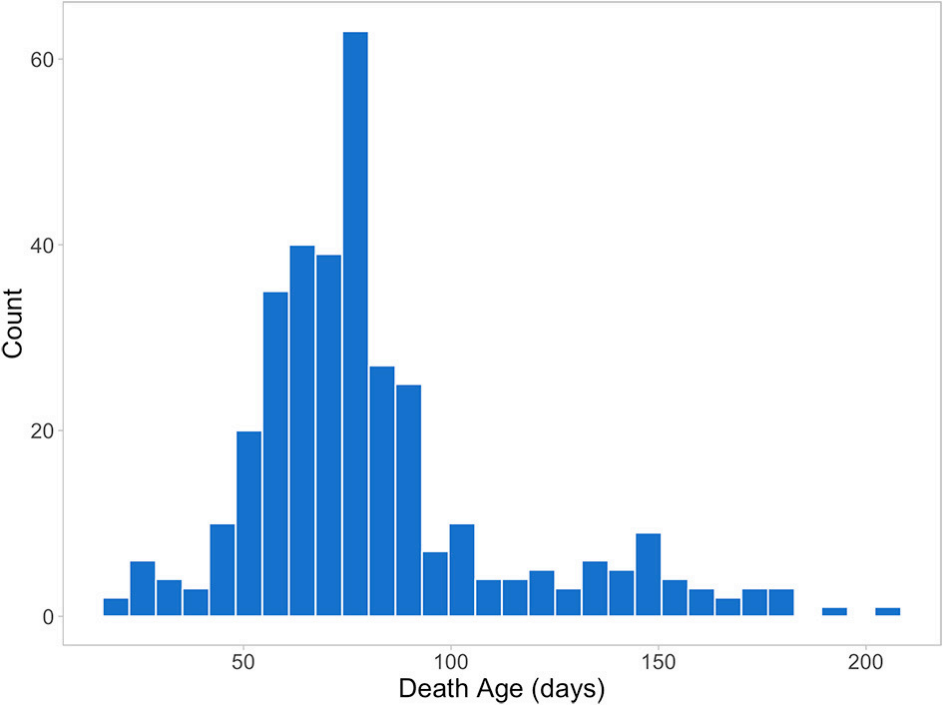
Age at death of the 344 animals that died prior to slaughter.

Figure 2 shows the distribution of death age for pigs that died prior to slaughter (344 or 26% of the 1,341 total animals). All treatment and mortality events and reasons were recorded (assigned by CDPQ research staff). Main treatment reasons included respiratory distress (thumping), gray/brown scours, coughing, lameness, yellow scours, arthritis, and failure to thrive/poor/skinny/hairy. Main mortality reasons included failure to thrive/poor/skinny/hairy, thumping/heavy breathing, sudden death, meningitis, and lameness/arthritis. Only individual treatments were included in the analyses and batch treatments were removed. Virtually all treatment reasons and ~89% of the mortality reasons were disease-related.

### Traits

Traits used for validation of the resilience traits developed herein included mortality (binary 0/1, 1 = died), number of treatments (**TRT**), and number of treatments per 180 days (**TRT180**). Number of treatments was a count of the number of individual treatments received by a pig. An individual treatment included any drug injection into an individual animal. Group treatments applied to batches were not included, as these would be accounted for in the model by the fixed effect of batch anyway. Only pigs that survived to slaughter received a phenotype for TRT. TRT180 was the number of treatments standardized to 180 days and was computed for animals that reached 65 days of age (approximate age of entry into the finishing unit). For instance, if an animal received three individual treatments and died on day 80, the animal's adjusted TRT180 was (3/80)^*^180 = 6.75. This was to standardize treatment rate to approximately the same scale as TRT and to be interpretable from a practical standpoint (number of treatments to slaughter).

**Figure 3:**
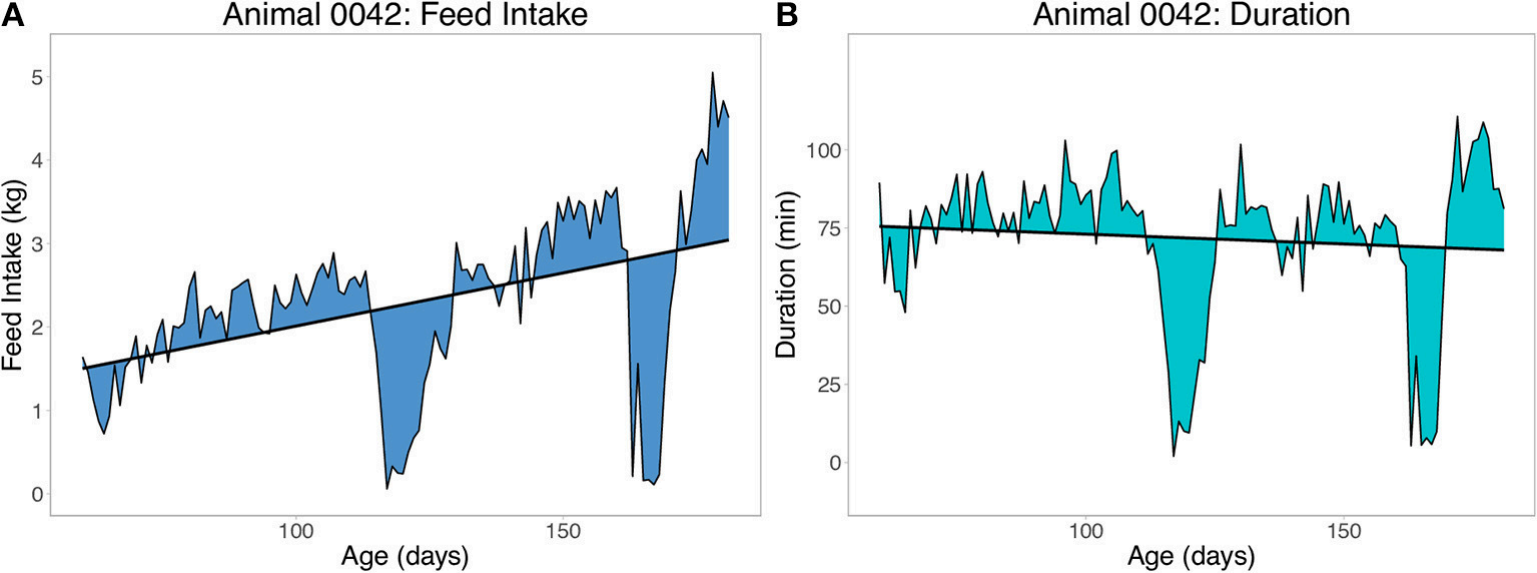
Example of the root mean square error (RMSE) of feed intake **(A)** and feeding duration **(B)** as measures of resilience. Duration is time spent at the feeder in minutes. Each animal received one record from a within animal regression of feed intake or duration on age.

Two sets of resilience traits were derived from the daily FI data available for each pig. The first set of traits were derived as the root mean square error (**RMSE**) within animal from the regression of feed intake (FI) or duration (DUR) on age (**RMSE**_**FI**_ and **RMSE**_**DUR**_, respectively), using ordinary least squares (OLS) linear regression. Duration is the daily time spent at the feeder in minutes. An example of the RMSE for one pig with two large deviations from illness is shown in Figure 3 for FI (Figure 3A) and duration (Figure 3B). To obtain a phenotype for RMSE, animals had to have a minimum of 60 days of FI recorded. A less resilient animal is expected to have a larger value for RMSE. Preliminary analyses showed that without setting this minimal number of days, animals that died early in finishing were grouped on the left side of the distribution of RMSE (i.e., they would be considered more resilient). Duration (time) at the feeder was chosen over traits such as number of meals due to its strong association with off-feed events (e.g., Figure 3).

**Figure 4:**
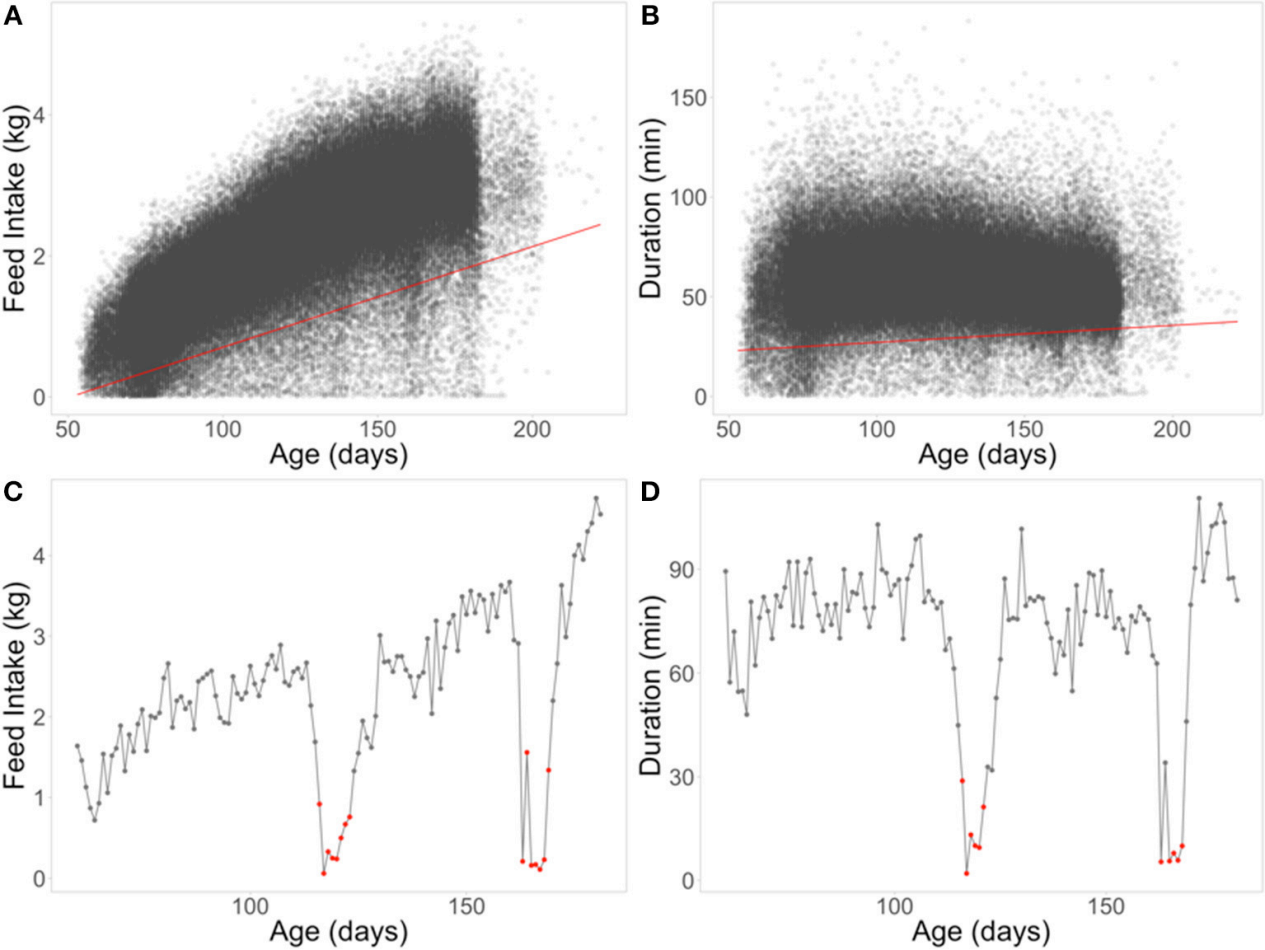
Quantile regression (QR) at the 5% level for feed intake **(A)** and duration **(B)**. Example of classifying off-feed days for animal 0042 using QR_FI_ **(C)** and QR_DUR_ **(D)** in the lower panels. Red lines are the 5% QR for each FI **(A)** and duration **(B)**. Red dots represent off-feed days used to calculate the QR phenotypes (proportion of off-feed days).

The second set of novel resilience phenotypes was based on quantile regression (**QR**), which can be useful for regression problems that include heterogeneous variances (Cade and Noon, 2003). A 5% quantile regression was performed using all data across batches, separately for FI and duration (Figures 4A,**B**). Negative residuals (below the regression line) from these regression equations were used to classify a day of FI or duration for an individual pig as an off-feed day (Figures 4C,D). These were aggregated within animal to a proportion of “off-feed” days (one record per animal). As with RMSE, each animal received only one phenotype for FI and for duration (**QR**_**FI**_ and **QR**_**DUR**_). The 5% threshold was set based on Figures 4A,B, as it separated the “cloud” of relatively healthy days from off-feed days, as well as appraisal of FI plots within animal. In total, 258 animals (25%) did not have any day below the 5% quantile regression for QR_FI_, while a 1% threshold resulted in 677 animals (65%) not having any days below the threshold. To obtain a phenotype for QR, animals had to have at least 60 days of FI recorded (same for RMSE). As with RMSE, susceptible animals are expected to have larger values for QR than resilient animals.

Production traits analyzed included nursery ADG (**NurADG**), finishing ADG (**FinADG**), average daily feed intake (**ADFI**), feed conversion ratio (**FCR**), residual feed intake (**RFI**), carcass weight (**CWT**), dressing proportion (**DRS**), lean yield (**LYLD**), carcass backfat (**CBF**), and carcass loin depth (**CLD**). To obtain a phenotype for a production trait, pigs had to complete the corresponding phase (nursery or finishing). Nursery and finishing ADG were calculated from regression slopes of daily LOESS weights (see above) on age for the entire nursery period (quarantine and challenge nursery) and the finishing period, respectively. NurADG started when the pig entered the quarantine nursery. NurADG ended and FinADG started the first day FI was recorded. LOESS predicted daily weights were used to compute ADG because a weight was not always available for the days when pigs were moved to the finishing unit. Also, some animals received more weights prior to being euthanized or death, which would influence the regression of weight on age (not evenly spaced). The impact of using LOESS predicted instead of observed weights was very small for FinADG (more weights) but was more significant for NurADG, as the correlation for FinADG with or without use of LOESS prediction was very high when using the closest endpoints, but much lower for NurADG. This was because the nursery period was much shorter, and many pigs only had two weights prior to being moved to the finishing unit and therefore a larger change in ADG was observed. Average daily feed intake (ADFI) was the average feed intake of daily records during the finishing period. Feed conversion ratio (FCR) was defined as the sum of daily records for FI over the total body weight gain for that same finishing period. Residual feed intake (RFI) was computed in a one-step analysis following Cai et al. (2008), using ADFI as the response variable and average body weight (average weight in the finisher), finishing ADG, and ultrasound backfat as covariates, along with other fixed effects, as described below. Ultrasound backfat was taken just prior to slaughter at the 10th rib. Dressing proportion was calculated by dividing the carcass weight (head on, leaf lard in, warm carcass) by the live weight prior to slaughter. Carcass backfat (CBF) and loin depth (CLD) were recorded using a Destron Fearing™ machine (Texas, USA) at the abattoir. Lean yield was calculated using the following regression equation for lean yield in Québec: LYLD = 68.1863 − (0.7833^*^CBF) + (0.0689^*^CLD) + (0.008^*^CBF^2^) − (0.0002^*^CLD^2^) + (0.0006^*^CBF^*^CLD) (Pomar and Marcoux, 2003). This equation was mostly driven by backfat (r = −0.98). Not all batches had carcass data, leading to some variation in the number of observations for these traits. Carcass phenotypes were also captured at different time points within batch due to the protocol to only send the pigs that met market weight at each slaughter date, as mentioned above. The average live weight at slaughter was 118.9 kg.

### Genotyping

Animals were genotyped with the 650 k Affymetrix Axiom Porcine Genotyping Array by Delta Genomics (Edmonton AB, Canada). In total, 658,692 single nucleotide polymorphisms (SNPs) were included on the chip. Raw Affyymetrix SNP data output was processed separately for each cycle by Delta Genomics with the Axiom® Analysis Suite using all defaults. The SNPs that passed quality control for all three cycles were utilized for analysis, for a total of 516,066 SNPs. Imputation of missing genotypes was completed with FImpute (Sargolzaei et al., 2014). The pedigree was utilized for imputation but only included the dam at the multiplier, since sire was typically unknown due to the use of pooled semen. Genotypes were then processed using the preGSf90 software from the BLUPF90 family of programs, using defaults (Misztal et al., 2002). Genotypes on seventeen samples were found to be duplicates and were removed. After all quality control, genotypes on 1,215 animals and 487,762 SNPs remained.

### Variance Component Analyses

Variance components were estimated by single-step GBLUP with the **H** matrix (Legarra et al., 2009; Christensen and Lund, 2010), using the BLUPF90 family of programs (Misztal et al., 2002). Data included phenotypes on 1,341 animals, of which 1,215 had genotypes. Basic animal models were fit for all traits, with random animal genetic effects (using the **H** matrix) and random residuals. The genomic relationship matrix (**G**) was calculated using **ZZ**′/sum(2pq) (VanRaden, 2008), where **Z** = **M**-**P**. Only the dam was available to construct the **A** matrix. Single trait models were used to obtain heritability estimates and bivariate models for genetic correlations. Models for mortality and number of treatments included batch and age of entry into the quarantine nursery as fixed effects and were modeled as linear traits. Mortality was initially analyzed as a threshold trait but resulted in unrealistically large estimates of heritability. A simulation was used to confirm that threshold models tended to significantly overestimate heritability with small sample sizes such as this study. One alternative could be to use a more recent approach from Ødegård et al. (2010) but mortality was not the main focus of this research. More data may be needed to analyze mortality as a threshold trait. Analyses for finishing traits included fixed effects of batch, finishing start age, and finishing pen. Litter effects (random) were minimal (below 0.05 for the proportion explained and within one SE of zero) for the traits analyzed and, therefore, were subsequently dropped from all analyses. Litter effects were also difficult to estimate, with an average of 2.02 litter mates per pig. Not all animals survived to record a phenotype for traits recorded later such as FinADG or carcass traits, therefore for these traits the average was < 2 litter mates per pig.

## Results

Table 1 shows summary statistics for the three cycles of data used in the analyses (seven batches per cycle). Batches included from 59 to 77 pigs, except for one batch of 28 (not shown), and each cycle ranged from 441 to 452 pigs (1,341 total). Mortality was highest in cycle one (35%), decreased in cycle 2 (13%), and then returned to a higher rate in cycle 3 (29%). Mortality per batch ranged from 4 to 57%, with the median being 18%. The continuous flow system maintained pathogen burden throughout the study, however, seasonality clearly led to higher mortality during the winter months. In contrast to TRT180, TRT did not follow the mortality trend due to the requirement of survival to slaughter. In general, the FI resilience phenotypes followed the same time trend as mortality, except for QR_DUR_.

**Table 1:** Counts and means for measures of resilience in three cycles of the natural challenge experiment (*n* = 1,341 total animals entered).

**Cycle^a^**	**Count**	**Mortality, %**	**TRT^a^**	**TRT180^b^**	**RMSE^c^_FI_, kg**	**RMSE^c^_DUR_, min**	**QR_**FI**_,^d^**	**QR_**DUR**_,^d^**
1	441	35	1.45	2.63	0.48	13.90	0.06	0.05
2	452	13	1.96	2.07	0.46	11.90	0.04	0.05
3	448	29	1.89	2.61	0.46	13.40	0.04	0.04

a *Number of treatments, animals must have made it to slaughter*.

b *Treatment rate adjusted to 180 days, animals must have made to through 65 days of age to obtain a phenotype*.

c *Root mean square error (RMSE) from the within animal regression of Feed Intake (FI) or Duration (DUR) on age with at least 60 days of FI*.

d *Quantile regression (QR) from using the 5% QR over all the feed intake (FI) or duration (DUR) data and then aggregating off-feed days within animal as a proportion*.

Table 2 shows the number of observations and summary statistics for each trait. RMSE and QR measures of resilience were required to have 60 days of FI to receive a phenotype, which removed 188 animals from those that made it into the finishing unit. The average RMSE_FI_ was 0.47 kg, ranging from 0.19 to 0.97 kg. RMSE_DUR_ averaged 13.10 min, with a range of 5.71 to 37.54 min. One major difference between TRT and TRT180 was that TRT180 allowed animals that died after 65 days of age to record a phenotype, which added 219 phenotypes. Of those that survived, the number of treatments was 1.79 on average, but 2.43 for TRT180 (median of 1.97). Due to the health challenges, many of the production phenotypes had a wide range. Nursery ADG ranged from 0 to 0.67 kg/d and finishing ADG from 0.36 to 1.20 kg/d. This caused carcass weights to have a wide range as well, despite the aim to slaughter at a “fixed weight.”

**Table 2:** Summary statistics of the analyzed traits (*n* = 1,341 total animals).

**Trait^a^**	**Number of phenotypes**	**Mean**	**SD**	**Median**	**Min**	**Max**
Mortality	1341	0.26^b^	N/A	0.18^c^	0.04^c^	0.57^c^
TRT^d^	997	1.79	1.56	1	0	10
TRT180^d^	1216	2.43	2.33	1.97	0	15.32
RMSE_FI_, kg	1036	0.47	0.11	0.45	0.19	0.97
RMSE_DUR_, min	1036	13.10	4.36	12.30	5.71	37.54
QR_FI_,	1036	0.04	0.07	0.01	0	0.67
QR_DUR_,	1036	0.04	0.06	0.02	0	0.52
NurADG, kg/day	1218	0.32	0.12	0.31	0.00	0.67
FinADG, kg/day	992	0.89	0.14	0.91	0.36	1.20
ADFI, kg/day	997	2.20	0.33	2.23	0.97	3.01
FCR, kg/kg	997	2.58	0.21	2.57	1.94	3.34
RFI, kg	991	N/A^e^			
CWT, kg	837	93.77	10.56	95.00	49.90	118.60
DRS,	837	0.78	0.02	0.78	0.68	0.84
LYLD,	799	60.92	1.71	60.90	55.20	65.60
CBF, mm	800	17.96	3.87	17.50	7.50	33.50
CLD, mm	800	60.69	6.14	60.50	41.50	81.00

a *TRT, number of treatments for animals that made it to slaughter; TRT180, treatment rate adjusted to 180 days for pigs that made it to 65 days of age; RMSE, root mean square error (novel phenotype with FI or duration); QR, quantile regression as a proportion (novel phenotype with FI or duration); NurADG, nursery ADG; FinADG, finishing ADG, ADFI, average daily feed intake; FCR, feed conversion ratio (kg feed / kg weight gain); RFI, residual feed intake (adjusted for FinADG, metabolic weight, and ultrasound backfat); CWT, carcass weight; DRS, dressing proportion; LYLD, lean yield (equation using backfat and loin depth); CBF, carcass backfat; CLD, carcass loin depth. RMSE and QR phenotypes required 60 days of FI*.

b *Overall mortality proportion*.

c *Median, min, and max by batch, not individual*.

d *TRT required the animal to survive to slaughter. TRT180 required the animal survive to 65 days of age*.

e *Residual feed intake (RFI) was calculated using ADFI as the response in a one-step method*.

Table 3 shows estimates of heritabilities and genetic correlations among the resilience traits and between resilience traits and production traits. Many estimates had large SE due to relatively small sample sizes. Heritability estimates for the novel resilience traits ranged from 0.15 to 0.26. Mortality had a heritability estimate of 0.13 ± 0.05, while TRT and TRT180 had estimated heritabilities of 0.13 ± 0.07 and 0.29 ± 0.07, respectively. The estimate of the genetic correlation between mortality and TRT180 was 0.93 + 0.29 (results not shown). Estimates of genetic correlations among the novel resilience measures ranged from 0.01 to 0.67, indicating they are different genetic traits. Estimates of genetic correlations of mortality and TRT180 with novel resilience traits were positive, as expected, and ranged from 0.37 to 0.85. Due to data processing and removal of phenotypes from TRT because of the requirement of survival to slaughter, TRT180 was deemed to be a better phenotype for validation of the novel traits (Table 2). The estimate of the genetic correlation of RMSE_DUR_ was 0.12 ± 0.76 with TRT and 0.62 ± 0.13 with TRT180. Of the two RMSE measures of resilience, RMSE_DUR_ was more highly correlated genetically with mortality and treatments than RMSE_FI_. For the QR traits, QR_FI_ had a slightly higher genetic correlation with mortality and number of treatments than QR_DUR_, which could be because farm staff received daily reports of which pigs were not eating enough feed and were flagged for further evaluation (see discussion).

**Table 3:** Estimates of heritability (SE) for traits analyzed and of genetic correlations (SE) with resilience measures (*n* = 1341 total animals, see Table 2 for actual counts per phenotype).

		**Genetic correlation (SE) with**			
**Trait^a^**	**h^**2**^**	**RMSE_**FI**_**	**RMSE_**DUR**_**	**QR_**FI**_**	**QR_**DUR**_**
RMSE_FI_, kg	0.21 (0.07)	–	0.47 (0.26)	0.50 (0.31)	0.52 (0.24)
RMSE_DUR_, min	0.26 (0.07)		–	0.67 (0.28)	0.01 (0.29)
QR_FI_,	0.15 (0.06)			–	0.64 (0.30)
QR_DUR_,	0.23 (0.07)	Symmetric			–
Mortality	0.13 (0.05)	0.37 (0.34)	0.60 (0.26)	0.75 (0.27)	0.70 (0.21)
TRT	0.13 (0.07)	0.52 (0.48)	0.12 (0.76)	0.76 (0.58)	0.62 (0.56)
TRT180	0.29 (0.07)	0.56 (0.18)	0.62 (0.13)	0.85 (0.16)	0.65 (0.15)
NurADG, kg/day	0.45 (0.07)	0.77 (0.24)	−0.10 (0.19)	−0.11 (0.25)	0.21 (0.20)
FinADG, kg/day	0.25 (0.07)	−0.31 (0.26)	−0.19 (0.26)	−0.75 (0.26)	−0.70 (0.17)
ADFI, kg/day	0.32 (0.07)	0.03 (0.26)	−0.24 (0.21)	−0.79 (0.19)	−0.58 (0.16)
FCR, kg/kg	0.35 (0.07)	0.39 (0.21)	−0.17 (0.25)	−0.14 (0.35)	0.02 (0.24)
RFI, kg	0.24 (0.07)	−0.22 (0.27)	−0.35 (0.25)	−0.78 (0.21)	−0.63 (0.16)
CWT, kg	0.31 (0.08)	−0.04 (0.28)	−0.13 (0.24)	−0.78 (0.25)	−0.63 (0.17)
DRS	0.10 (0.06)	−0.23 (0.07)	−0.49 (0.49)	−0.73 (0.60)	−0.52 (0.53)
LYLD,	0.50 (0.08)	0.13 (0.24)	0.00 (0.23)	0.50 (0.24)	0.37 (0.19)
CBF, mm	0.46 (0.09)	−0.14 (0.26)	0.03 (0.23)	−0.36 (NA)	−0.35 (0.18)
CLD, mm	0.39 (0.08)	−0.20 (0.27)	−0.05 (0.24)	−0.29 (0.30)	−0.21 (0.25)

a *RMSE, root mean square error (for FI or duration); QR, quantile regression as a proportion (for FI or duration); TRT, number of treatments for animals that made it to slaughter; TRT180, treatment rate adjusted to 180 days for pigs that made it to 65 days of age; NurADG, nursery ADG; FinADG, finishing ADG; ADFI, average daily feed intake; FCR, feed conversion ratio (kg feed / kg weight gain); RFI, residual feed intake (adjusted for FinADG; metabolic weight; and ultrasound backfat); CWT, carcass weight; DRS, dressing proportion; LYLD, lean yield (equation using backfat and loin depth); CBF, carcass backfat; CLD, carcass loin depth. RMSE and QR phenotypes required 60 days of FI*.

Estimates of genetic correlations of RMSE traits with production traits were low, but many were favorable (Table 3). Nursery ADG was unfavorably correlated with RMSE_FI_ (0.77 ± 0.24) but most of the other production traits had favorable or close to zero genetic correlations with the two RMSE measures of resilience. Finishing ADG had a genetic correlation estimate of −0.31 ± 0.26 with RMSE_FI_ and of −0.19 ± 0.26 with RMSE_DUR_. Feed efficiency based on FCR and RFI were genetically correlated with RMSE_FI_ (0.39 ± 0.21 and −0.22 ± 0.27, respectively). Resilience based on QR measures was more strongly associated with production traits than resilience based on RMSE. Both QR_FI_ and QR_DUR_ had strong genetic correlations with FinADG, at −0.75 ± 0.26 and −0.70 ± 0.17, respectively. Notice, however, that QR was not strongly correlated with NurADG, likely because feed intake was only collected in the finisher. ADFI was negatively correlated with QR_FI_ and QR_DUR_, at −0.79 ± 0.19 and −0.58 ± 0.16, respectively. Estimates of genetic correlations of QR with FCR were low, at −0.14 ± 0.35 and 0.02 ± 0.24, vs. −0.78 ± 0.21 and −0.63 ± 0.16 with RFI, which were similar to those for ADFI. Carcass BF and LD had negative genetic correlations with QR measures of resilience (−0.36 to −0.21).

Table 4 shows estimates of genetic correlations of production traits with mortality and number of treatments. Production traits tended to have low genetic correlations with mortality (< 0.30 in absolute value) but higher with number of treatments for some traits. Estimates of the genetic correlation of finishing ADG and ADFI with TRT and TRT180 ranged from −0.60 to −0.70. Carcass weight also showed a strong negative genetic correlation of −0.67 ± 0.14 with TRT180, similar to FinADG. Carcass BF, LD, and LYLD were weakly genetically correlated with both number of treatments and mortality.

**Table 4:** Estimates of genetic correlations (SE) of mortality and number of treatments with production traits.

**Trait^a^**	**Mortality**	**TRT**	**TRT180**
NurADG, kg/day	0.27 (0.44)	−0.33 (1.28)	−0.06 (0.16)
FinADG, kg/day	−0.06 (0.36)	−0.68 (0.42)	−0.70 (0.13)
ADFI, kg/day	−0.04 (0.28)	−0.60 (0.32)	−0.62 (0.13)
FCR, kg/kg	0.24 (0.28)	−0.15 (0.43)	0.13 (0.18)
RFI, kg	−0.29 (0.31)	−0.41 (0.45)	−0.53 (0.19)
CWT, kg	0.02 (0.33)	−0.57 (0.36)	−0.67 (0.14)
DRS,	dnc^b^	dnc^b^	−0.63 (0.35)
LYLD,	0.01 (0.34)	−0.14 (0.40)	−0.01 (0.20)
CBF, mm	dnc^b^	0.17 (0.48)	0.01 (0.21)
CLD, mm	0.27 (0.33)	−0.04 (0.38)	−0.12 (0.20)

a *NurADG, nursery ADG; FinADG, finishing ADG; ADFI, average daily feed intake; FCR, feed conversion ratio (kg feed/kg weight gain); RFI, residual feed intake (adjusted for FinADG, metabolic weight, and ultrasound backfat); CWT, carcass weight; DRS, dressing proportion; LYLD, lean yield (equation using backfat and loin depth); CBF, carcass backfat; CLD, carcass loin depth*.

b *Did not converge*.

## Discussion

Novel disease resilience measures were extracted from daily feed intake data of grow-finish pigs that were exposed to a multifactorial natural disease challenge that was designed to mimic a commercial environment with high disease pressure to maximize the expression of genetic differences of resilience between animals. Although the specific disease and environmental conditions that were established in this study cannot be exactly replicated, the general protocols established can be replicated in both research and commercial settings, similar to the replication of field studies on health-challenged farms. Moreover, although infection pressure waxes and wanes over time, it is assumed to be relatively consistent within batch because of the close proximity in which new batches are housed.

The resilience traits that were derived from individual daily feed intake data showed moderate heritabilities and moderate to strong genetic correlations with mortality and treatment rate. Genetic correlations production traits tended to be low for the RMSE measures of resilience but higher for the QR measures. Data from the most important disease exposure period, i.e., the challenge (2nd) nursery, were not included in either RMSE or QR measures of resilience because individual feed intake could only be collected in the finishing unit. The challenge nursery period was, however, critical, as this represented the first exposure to many pathogens in the barn for most batches (nose-to-nose contact for new batches with older already infected batches). Thus, pigs could have been infected with pathogens and recovered in the nursery before feed intake recording started in the finishing unit. This may have reduced genetic correlations of the evaluated novel resilience traits with mortality or number of treatments. Future research could address this by collecting important phenotypic data during the entire challenge period or by setting up the nursery away from the finishing challenge facility.

The RMSE measures of resilience proposed here were designed to quantify severity of disease and other stressors on individual animals over time (see below), whereas QR measures of resilience classified days as off-feed events, reflecting more extreme events, making the QR measures less sensitive and showing less variation than RMSE measures. This may partially explain the slightly lower estimates of heritability for QR compared to RMSE measures and the higher genetic correlations of QR with TRT180 and mortality than RMSE, as both mortality and treatments are the result of severe clinical disease. Pigs were typically not euthanized until the disease had progressed and the animal was clearly suffering. Treatments were generally given only when clinical signs of illness were present (e.g., diarrhea, coughing, lethargy, etc.). RMSE measures of resilience may have the ability to capture subclinical disease and other stresses in addition to clinical disease, enabling it to be more sensitive than number of treatments, mortality, and QR measures of resilience, which typically capture only severe events. This would make RMSE measures of resilience different traits than treatments, mortality, and QR, which was supported by the estimates of genetic correlations. Although QR measures of resilience can also capture the effects of stressors other than disease, it is less likely to do so compared to RMSE due to the larger impact of disease on feed intake compared to other stressors (results not shown).

Quantile regression measures of resilience tended to have higher genetic correlations with production traits than RMSE, likely because pigs that grow slower typically have lower ADFI and, thus, when they get sick, they need a smaller drop in FI to drop below the QR line. In contrast, animals with high ADFI must drop further to have a drop below the QR threshold. Thus, pigs with low average FI across the finishing period are expected to have more days classified as being off-feed days, resulting in higher genetic correlations of QR measures of resilience with traits that are closely related to FI such as ADG, than RMSE. Refining these resilience phenotypes will be a focus of future research.

### Feed Intake Duration

Feeding duration was used in this study as a proxy for drops in FI. In the past, there have been many attempts to link feeding traits with FI (de Haer et al., 1993; Von Felde et al., 1996; Young et al., 2011; Lu et al., 2017). In animal breeding, these traits include duration (time at the feeder), number of visits, and feed intake rate. Previous studies were typically conducted in healthy environments and feeding traits such as duration at the feeder may become more valuable under disease challenge. Figures 3, 4 show how the pattern of FI and duration were very similar across time for this selected animal. Measures of resilience based on duration had comparable genetic correlations with mortality and number of treatments as measures of resilience based on FI in the present study. Day-to-day variation in duration at the feeder could be more applicable on commercial farms if commercial feeders could be retrofitted to record individual time at the feeder using antennae and RFID tags. This could also be extended into the nursery, allowing feeding traits to be collected over the entire wean-to-finish period. Additional research is needed to evaluate other feeding traits that can be extracted from electronic feeders (Kyriazakis and Tolkamp, 2018). Feeding patterns within a day may be useful and could be utilized to better quantify resilience. The current study took the simple approach and used daily totals, but this is only a starting point for more research on this topic.

### Causes of Variation in FI and Their Relationship With Resilience

Colditz and Hine (2016) presented a holistic view of resilience by including other stressors to define general environmental resilience. In the current study, it is not possible to verify that all drops in FI and duration at the feeder observed in our data are due to disease alone. Martínez-Miró et al. (2016) categorized animal stressors into social, environmental, metabolic, immunological, and human interactions. Each of these could be decomposed into more detailed stressors. For instance, immunological stressors can be broken down further into individual resistance, tolerance, or resilience toward PRRSV or PCV2 (among others). There can also be interactions between these stressors (Salak-Johnson and McGlone, 2007), although other studies have suggested some stressors may be additive (Hyun et al., 1998).

There is a long list of stressors that can impact feed intake and performance on swine. The impact of pathogens on feed intake has been well established in the literature (Sandberg et al., 2006; Kyriazakis and Doeschl-Wilson, 2009) and is dependent upon, but is not limited to, the type of pathogen, the strain of the pathogen, previous exposure, and vaccinations. Porcine reproductive and respiratory syndrome virus alone costs the swine industry an estimated $664 million annually in the US (Holtkamp et al., 2013). Heat stress is another common reason why animals deviate from their expected FI (Guy et al., 2017), which has been characterized in growing pigs (Rauw et al., 2017b) and in sows (Vilas Boas Ribeiro et al., 2018). Mycotoxins have been known for a long time to influence feed intake (Smith et al., 1997). Social interactions (i.e., space requirements) are another common source of stress in swine (Hyun et al., 1998). These social effects have been investigated in piglets (Bouwman et al., 2010), in growing pigs (Street and Gonyou, 2008), as well as in sows (Hemsworth et al., 2013). Martínez-Miró et al. (2016) discussed many other stressors including human handling, vaccination, dust/gas/ammonia, and out of feed and water events.

Knap (2009) originally used an example of heat stress in pigs to show the potential relevance of day-to-day variability in feed intake. The measures of resilience developed here could also be used to quantify resilience to heat tolerance (Fragomeni et al., 2016; Guy et al., 2017), activity level (Sadler et al., 2011; Gilbert et al., 2017; King et al., 2018), and possibly even reduce stressful interactions for pigs (Rauw et al., 2017a). Heat stress was estimated to cost the US swine industry $299 million per year (St-Pierre et al., 2003). These measures could also be based on other sources of data such as water intake data (Madsen and Kristensen, 2005; Rusakovica et al., 2017) or body temperature recordings on individual pigs (Petry et al., 2005, 2017). Elgersma et al. (2018) developed variation and “drop phenotypes” from milk yield data in dairy cows. The phenotypes developed in the current study could also be used to develop similar phenotypes for other species.

A problem with the interpretation of the types of resilience measures developed here and by Elgersma et al. (2018) is that factors influencing resilience phenotypes in general are still a “black-box” (Mulder and Rashidi, 2017), not only in terms of different diseases but for all the other stressors described above. This is one reason why we cannot expect the genetic correlation between RMSE and mortality or treatments to be one, as factors that influence feed intake, could be non-health related. Another reason may be that RMSE captures sub-clinical disease better than QR (Elgersma et al., 2018 mentions this also for their resilience traits). Although from a practical or commercial breeding standpoint, it probably matters little why animals deviate from expected feed intake. Traits presented in the current study should be thought of as having economic value (Elgersma et al., 2018). The usefulness of these novel traits in a breeding program will depend on the commercial environment and how representative the testing herds are of the target environments.

### Genetic Parameters

Most estimates of heritability for production traits were within the accepted industry range (Ciobanu et al., 2011; Clutter, 2011), although this study was conducted under a strong health challenge. To the best of our knowledge, there are no estimates of genetic parameters for the novel resilience traits evaluated here in pigs. Variation for different traits has been explored as a potential indicator trait for resilience in dairy cattle. Green et al. (2004) evaluated the use of changes in somatic cell count (SCC) over time as an indicator for mastitis in lactating dairy cows and concluded that the maximum SCC and the standard deviation of log SCC were the best phenotypic indicators for incidence of mastitis. Recently, Elgersma et al. (2018) estimated genetic parameters for resilience traits from daily milk yield data from automated milking systems. Resilience indicators from milk yield data were calculated using the sum of “drop” days, negative slopes, and overall variation in milk yield calculated within lactation for each cow. Heritability estimates ranged from 0.06 to 0.10 and genetic correlations of variation in milk yield with udder health, ketosis, longevity, and persistency ranged from −0.29 to −0.52 (Elgersma et al., 2018). Elgersma et al. (2018), however, did not account for the individual cow milk yield trajectory over lactation when computing day-to-day variation in milk yield but targeted this for future research.

Heritability estimates for mortality and treatments in pigs are difficult to find in literature because of the swine pyramid structure, which results in most studies focusing on data collected in herds with limited disease. Guy et al. (2018) estimated the heritability of treatments to be between 0.04 and 0.06. Commercial test herds using the three-way terminal cross are becoming more popular in the swine industry but results from such data are not commonly reported in the literature. One example is Dufrasne et al. (2014), who used a sire model to estimate variance components for mortality (culling) traits. Heritability estimates ranged from 0.03 to 0.14 using threshold models (Dufrasne et al., 2014) but the rate of mortality after weaning was very low (< 1%), which seems very unrealistic as typical commercial wean-to-finish barns have between 6 and 9% mortality on average (Stalder, 2017). Estimates of heritability for treatment and mortality could change with the amount of health challenge and incidence (Bishop and Woolliams, 2014). Companies will need to decide how much of a health challenge they need if they aim to select for resilience to disease. Challenging pigs too much comes at an economic and animal welfare cost. If not challenged enough, heritabilities of mortality, and treatments may become lower and response to selection will be slowed (Mulder and Rashidi, 2017), although low heritabilities may be partially overcome with very large family sizes (many matings per sire). Treatment data is also challenging to collect in commercial testing systems. Many use mass treatments for disease outbreaks (e.g., feed and water medication). Water treatments may be more helpful for treatment under challenge, while feed medications may be more helpful for prevention (due to the off-feed events under challenge). Although it is possible to collect individual treatment data, commercial farms differ in the amount of data, and details they record. Factors such as withdrawal times may influence when and if an animal receives treatment. When animals are treated and/or euthanized is based in part on subjective decisions by farm or veterinary staff. If antibiotic free production is involved, this may also influence the decision to treat an animal or not. Resilience is expected to be more economically important in those conditions as management cannot mask the genetic potential for resilience.

### Implementing Quantile Regression (QR) Phenotypes

One of the challenges when implementing QR phenotypes is that the quantile regression equation will depend on the severity of the disease challenge. For instance, if one barn is completely healthy over the years and another barn is severely challenged, the QR equation for each barn will be very different even if both are at the 5% level. This difference will be tied to how often contemporary groups are challenged and to what degree they are challenged. Mulder and Rashidi (2017) discussed the percentage of contemporary groups challenged and how that affects selection efficiency. When starting a commercial testing system, setting this initial QR threshold may be difficult. If the first groups are not heavily challenged, it will lead to setting the QR equation too high and capturing days that are not due to illness and other stressors, simply normal daily variation in FI. With a weaker disease challenge, a more appropriate QR may be 1%. One possibility is to create a training dataset for QR based on contemporary groups that were challenged and set the threshold based on that regression. Another possibility would be to take only healthy contemporary groups and set a lower bound threshold based on that data.

### Heterogeneous Residual Variance in FI Data

One problem with using daily variation in FI vs. duration is that the variance of FI increases with age, which is not observed for daily duration data. This results in stressors having a greater impact on FI for older vs. younger pigs. As a result, RMSE_FI_ puts a greater weight on later compared to earlier ages. Mean duration showed a slight negative trend with age and its variance was fairly constant across the finishing period. This may be one reason for the fairly low genetic correlation between FI and duration measures and could also explain why RMSE_DUR_ had slightly higher genetic correlations with mortality and number of treatments than RMSE_FI_, as RMSE_DUR_ weights the early finishing period the same as the late finishing period. An attempt was made to adjust RMSE_FI_ for this increasing variation over time, but this still resulted in large outliers at later ages and did not improve the phenotype much in terms of genetic correlations to mortality and treatments (results not shown).

### Causes of Mortality

Most recorded mortality reasons were linked to disease. Exceptions included death from blood sampling, rectal prolapse, fighting, and fracture/sprains, which amounted to ~11% of the 344 mortalities observed in these data. Removing mortality records due to non-health reasons, however, only resulted in small changes in estimates of heritability and genetic correlations, so they were left in the dataset for the current analyses, also because mortality by definition includes any pig that died regardless of cause. Although we typically think of mortality as health related, it is very multifactorial, as is sow mortality. One could decide to separate mortality by cause due to different genetic architecture for each cause and different economic weights (due to the average timing of death), but it is likely that the heritability would be even lower due to lower incidence, which would limit genetic selection. In addition, mortality for non-health related reasons could also have a genetic component. Treatments were almost exclusively linked to disease. Although some, such as lameness, could be argued to not be linked to disease, some diseases can be linked to lameness and removing them then becomes controversial.

### Impact of Increased Variation in Performance

One major impact of disease is the increase in variation in production phenotypes such as growth, causing some pigs to be less than full value when harvested (Fix et al., 2010). In the current study, if pigs did not make weight, they were held in the finisher until they made the target weight range, resulting in more pigs achieving full value when harvested. The definition of full value is not consistent in research or the swine industry and was therefore avoided in this analysis. Some production systems require all animals to leave at a certain date regardless of weight (i.e., fixed time systems). This would lead to additional costs from disease as a result of greater variability in slaughter weights and carcass weights. Hubbs et al. (2008) used moments beyond the mean to include variance and skew for determining optimal marketing decisions and concluded these higher-order statistics appeared to be more important than they were in the past. Not only are carcasses lighter and therefore worth less in total, sort loss (or discount losses) from not meeting the optimal weight grid will also penalize these animals (Boys et al., 2007). Sometimes, these lightweight animals can go to alternative markets, but not always (Fix et al., 2010).

### Use of Novel Traits in Healthy Nucleus Environments

The novel traits evaluated here can also be recorded in relatively healthy nucleus environments for stressors other than health challenges. A second major factor impacting these novel traits may be heat stress. The genetic correlation between RMSE in the nucleus vs. in a commercial environment (under disease challenge) will likely depend on the level and nature of the disease challenge in the commercial environment and the amount of heat stress in each environment (among other stressors). Barns located in the Southern/SE USA region will be affected differently by heat stress than those in the upper Midwest or Canada. This barn was in Québec, Canada, and the heat stress experienced was minimal compared to other areas around the world. The novel resilience traits evaluated here will likely have lower means and be less variable and have lower heritability under nucleus conditions but will still include resilience to stressors.

## Conclusions

Day-to-day variation (RMSE and QR) in feed intake or duration at the feeder can be used to quantify resilience in health challenged environments, such as a commercial testing scheme. The novel resilience phenotypes studied here were moderately heritable and genetically correlated with mortality and treatment rate. The genetic correlations reported here may underestimate true correlations because the initial challenge period was missed because pigs were first challenged in the nursery and feed intake data for RMSE and QR was recorded in the finishing unit only, while mortality and treatments were recorded over the entire wean-to-finish period. Many factors can cause variation in feed intake and in time at the feeder, including disease, heat stress, handling, and social interactions. Thus, the measures of resilience investigated here are still “black-box” phenotypes and should be viewed as general resilience instead of the narrower concept of disease resilience. Overall, daily variation in FI or associated duration data can be used to quantify resilience.

## Author Contributions

AP came up with the novel phenotypes, analyzed the data, and wrote the manuscript with help from JD. GP, MD, PGC, JH, FF, and JD designed the project and developed protocols for animal sourcing, management, and phenotype recording. JH was in charge of veterinary oversight on the project. GP was in charge of the database and genotyping for the project. FF was the lead on day-to-day data collection and scheduling. All authors helped with interpretation of the results and reviewed and approved the final manuscript.

### Conflict of Interest Statement

The authors declare that the research was conducted in the absence of any commercial or financial relationships that could be construed as a potential conflict of interest. The handling Editor declared a past co-authorship with one of the authors JD.
